# A Geometric Analysis of Polyethylene Liners Exposed to Acrylic-based Bone Cement

**DOI:** 10.1016/j.artd.2023.101184

**Published:** 2023-09-19

**Authors:** Zoe Thompson, Harry Hothi, Jacqueline Brillantes, Amir Khoshbin, Amit Atrey

**Affiliations:** a University of Toronto, Toronto, Canada; b The Royal National Orthopaedic Hospital, Stanmore, UK; c St. Michael’s Hospital, University of Toronto, Toronto, Canada

**Keywords:** Hip arthroplasty, Geometric analysis, Polyethylene, Acetabular component, Acrylic-based bone cement

## Abstract

**Background:**

Acrylic-based bone cement (polymethyl methacrylate [PMMA]) is a material commonly used in orthopaedic surgeries; however, during PMMA polymerization, a highly exothermic reaction occurs. The heat released in polymerization can damage nearby materials including poorly heat-resistant cross-linked polyethylene (XLPE). Both PMMA and XLPE are used in total hip arthroplasty and could interact during femoral stem fixation. We sought to determine if the exothermic polymerization of PMMA could alter the surface characteristics of XLPE acetabular liners.

**Methods:**

Six XLPE liners were assigned to one of 4 experimental categories with varying volumes of PMMA applied in a manner that mimicked how the 2 materials would come into contact intraoperatively. Measurements were taken both pre- and post-intervention using a coordinate measuring machine for geometric and gravimetric analysis. Light microscopy was conducted postintervention to examine the surface for damage.

**Results:**

Coordinate measuring machine measurements showed minimal gross deformation in all 6 liners, but there were isolated surface deposits in 4 of 6 liners. The average maximal surface deviations, when compared to the control, for liners exposed to 1 cc of cement, 2 cc of cement, or 1 cc of cement with a femoral head implant attached were 26.6 μm, 77.2 μm, and 26.4 μm, respectively. All but one liner showed an increase in volume following intervention when compared to the control. Subtle scratches were identified using light microscopy on all 6 liners.

**Conclusions:**

XLPE shows areas of isolated surface deformation in a dose-dependent manner but with minimal gross deformation after interacting with highly exothermic PMMA.

## Introduction

Acrylic-based bone cement (polymethyl methacrylate [PMMA]) is frequently used in orthopaedic surgeries [1] for fixation of prostheses including those in total hip arthroplasty (THA) [2]. The success of cemented hip prostheses is well documented [3].

However, there are negative factors that should be considered when using bone cement including its thermal properties [4]. One disadvantage of the preparation of bone cement is that it creates an exothermic reaction during polymerization resulting in the release of energy to its surroundings [4]. This energy transfer is highly exothermic, with temperatures extending as high as 113°C. It has been observed to deteriorate surrounding structures such as bone tissue [5].

Efforts have been made to improve the thermal performance of PMMA, including adding different nanoparticles such as magnesium oxide, hydroxyapatite, chitosan, and silica [6]. In addition to the potential to deteriorate surrounding bone, PMMA may have detrimental effects on other materials used in surgical procedures. In the case of THA, cross-linked polyethylene (XLPE) is a common material used in acetabular liners. Polyethylene itself has poor resistance to heat and shows deformation at 100°C and melting at 130°C. [7].

Although bone cement is not intended to come into contact with the articulating surface of polyethylene liners intraoperatively, the 2 may inadvertently do so during femoral stem fixation. As the liquid cement is placed into the joint, it can inadvertently extrude from the canal or the cement and land in the cup. Although cement is removed prior to full hardening, the liquid form is still highly exothermic and can impart heat in surrounding structures prior to removal.

Observing how the surface of a polyethylene liner would react to the exothermic process of PMMA polymerization and hardening will provide a greater understanding of any resulting polyethylene deformity, that could result in increased wear and potential for early THA failure [8]. This would be critical to determine if greater precautions are required to be in place should the articulating surface of a polyethylene liner be exposed to polymerizing cement during THA.

The aim of this study was to assess any damage to polyethylene liners after contact with actively polymerizing bone cement, as may happen inadvertently during THA. To achieve this, we conducted geometric analysis of polyethylene liners before and after contact with cement in a manner that would mimic how the 2 materials would come into contact intraoperatively.

## Material and methods

### Cement application

A total of six polyethylene liners were part of the study in a total of 4 experimental conditions (Table 1). All liners were the same size (54 mm) with an aperture for a 32 mm head and were all made of X3 XLPE (Stryker, Mahwah, NY). Simplex polymethyl methyl acrylate (Marwah, USA – Stryker) cement was measured in liquid state using a standard syringe and then was injected into the bearing surface of the polyethylene liner while still liquid. The cement was left on the liner for 16 minutes, such that the cement had completely polymerized and hardened. This both mimics what would happen intraoperatively as well as ensuring the full exothermic reaction has occurred to impart maximal thermal stress on the polyethylene. The cement was removed using digital sweep, again to replicate what would be done by the surgeon intraoperatively, with fine-tooth forceps used to remove additional debris to minimize damage to the polyethylene component. Differing amounts of cement were placed, and in one liner, a femoral head was placed to mimic what might happen if the cement were missed and the head was reduced during the implant trial. The volumes used (Table 1) were significantly less than the total amount of cement typically used intraoperatively, again to mimic the scenario of inadvertent extrusion of portions of cement onto surrounding materials.

**Table 1:** Experimental conditions for the 6 polyethylene liners.

Liner number	Amount of cement injected onto liner surface (cc)	Liner environment
1	1	Alone
2	1	Alone
3	2	Alone
4	2	Alone
5	1	With a femoral head implant attached
6 (control)	0	Alone

### CMM scans

In the analysis of the 6 Trident X3 polyethylene liners (Stryker, Mahwah, NY), a Zeiss Prismo (Carl Zeiss Ltd., Rugby, UK) coordinate measuring machine (CMM) was utilized to capture their articulating surface geometry in the form of a point cloud [9]. The CMM’s 2 mm ruby contact stylus was instructed to perform meridian arcs of longitude along each surface, which emanated from the pole and terminated within 1 mm of the rim. In accordance with ISO 14242-2 and ASTM F2979 standards, these arcs were separated by a maximum distance of 0.5 mm at the equator, while a point separation (point pitch) of 0.1 mm was adopted along each arc.

These CMM scans were performed prior to and following experimentation, allowing the assessment of surface changes. The captured point clouds were triangulated to form a surface model. Within a commercially available software (Simpleware Scan IP, Synopsis, Exeter, UK), both surfaces were registered to enable the visualization of any surface deviations.

Deviations greater than 20 μm and within the range of ±0140 μm were mapped to allow a comparison between all samples. The range of surface deviations was included in the color map and optimized for each sample. The maximum and minimum surface deviations are stated in each case, in addition to notable deviations identified in each map.

A validated method for quantifying material volume change from the articulating surfaces of hip implants was adopted to assess their geometry following experimentation [10,11]. The liners were found to deviate from a perfect sphere in their pristine state, and this hindered the ability to reliably report the volumetric change that had occurred following experimentation. Consequently, a gravimetric approach was adopted to provide a measure of any volumetric changes that had occurred following experimentation. Using this approach, the mass of the liner was taken pre- and post-experimentation, and volume loss was calculated based on a polyethylene density of 0.0,009,408 g/mm.

### Microscopy

A Keyence VHX-700F (Keyence Co., Japan) light microscope was used to examine the surfaces of the liners after removal of cement. Magnifications of 20×-200× were used to identify any signs of damage.

## Results

### Geometric analysis

**Figure 1:**
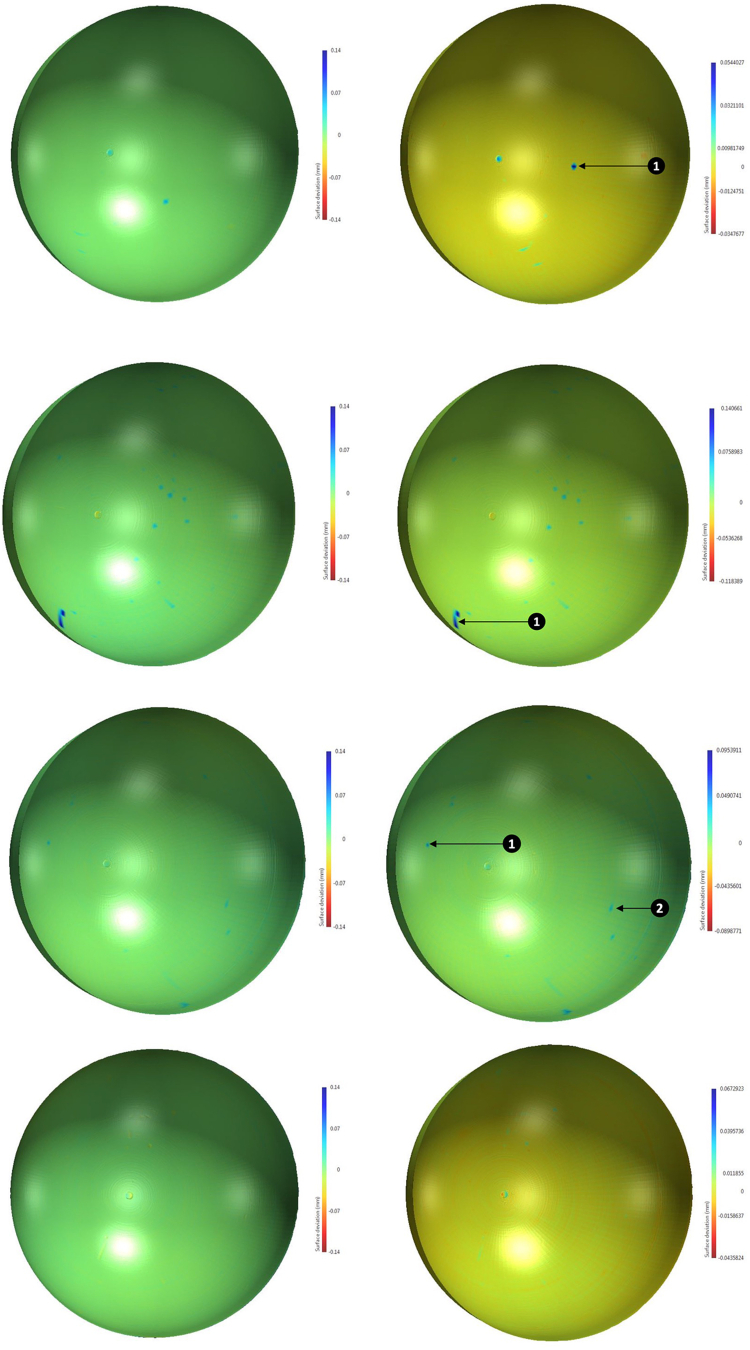
Heat mapped surfaces of the polyethylene liners based on pre- and post-experimentation measurements (top to bottom: liner 1, liner 3, liner 4, liner 5). Isolated areas of deposit are marked with arrows and corresponding numbers.

On geometric analysis, there was minimal evidence of gross deformation (widespread thinning across the liner) on all 6 liners; however, in four of the 6 liners, isolated areas of deformation were noted, the largest of which extended 140.7 μm above the surface of the liner. Liner 2 and 6 (control) did not show any distinct surface deposits. Any areas of notable surface deviation or deposit were marked with a number and/or heat mapped (Fig. 1). Liner 4 did not have any large singular deposits but did have a cluster of deposits that could represent an area of interest and so it is included in this group. Measurements of both the upper and lower surfaces were taken, and maximum deviations from pre-experimental scanning were determined (Table 2). The average maximal surface deviations, when compared to the control, for liners exposed to 1 cc of cement, 2 cc of cement, or 1 cc of cement with a femoral head implant attached were 26.6 μm, 77.2 μm and 26.4 μm respectively.

**Table 2:** CMM derived maximum surface deviation on both upper and lower surfaces.

Liner number	Upper surface maximal deviation (μm)			Lower surface maximal deviation (μm)		
	Computed result	Control comparison	Average	Computed result	Control comparison	Average
1	54.4	13.5	26.6	−34.8	30.9	25.1
2	80.5	39.6		−46.5	19.2	
3	140.7	99.8	77.2	−118.4	−52.7	−38.5
4	95.4	54.5		−89.9	−24.2	
5	67.3	26.4	-	−46.3	19.4	-
6	40.9	-	-	−65.7	-	-

### Gravimetric analysis

The mass of the polyethylene liners was measured both before and after cement application in order to assess change in volume. The density of polyethylene used in these calculations was 0.0,009,408 g/mm. The average amount of material volume change was 1.15 mm^3^ with values ranging from −2.02 mm^3^ to 2.02 mm^3^ across the 6 liners (Table 3). When compared to the control condition, all liners increased in volume following cement application and subsequent removal with the exception of liner 2.

**Table 3:** Volume change determined by pre- and post-experimentation gravimetric analysis.

Liner number	Volume change (mm^3^)	Volume change compared to control (mm^3^)
1	1.95	0.43
2	−2.02	−3.54
3	1.84	0.32
4	1.56	0.04
5	2.02	0.50
6	1.52	
Average	1.15	−0.45
Range	4.04	

### Microscopic imaging

**Figure 2:**
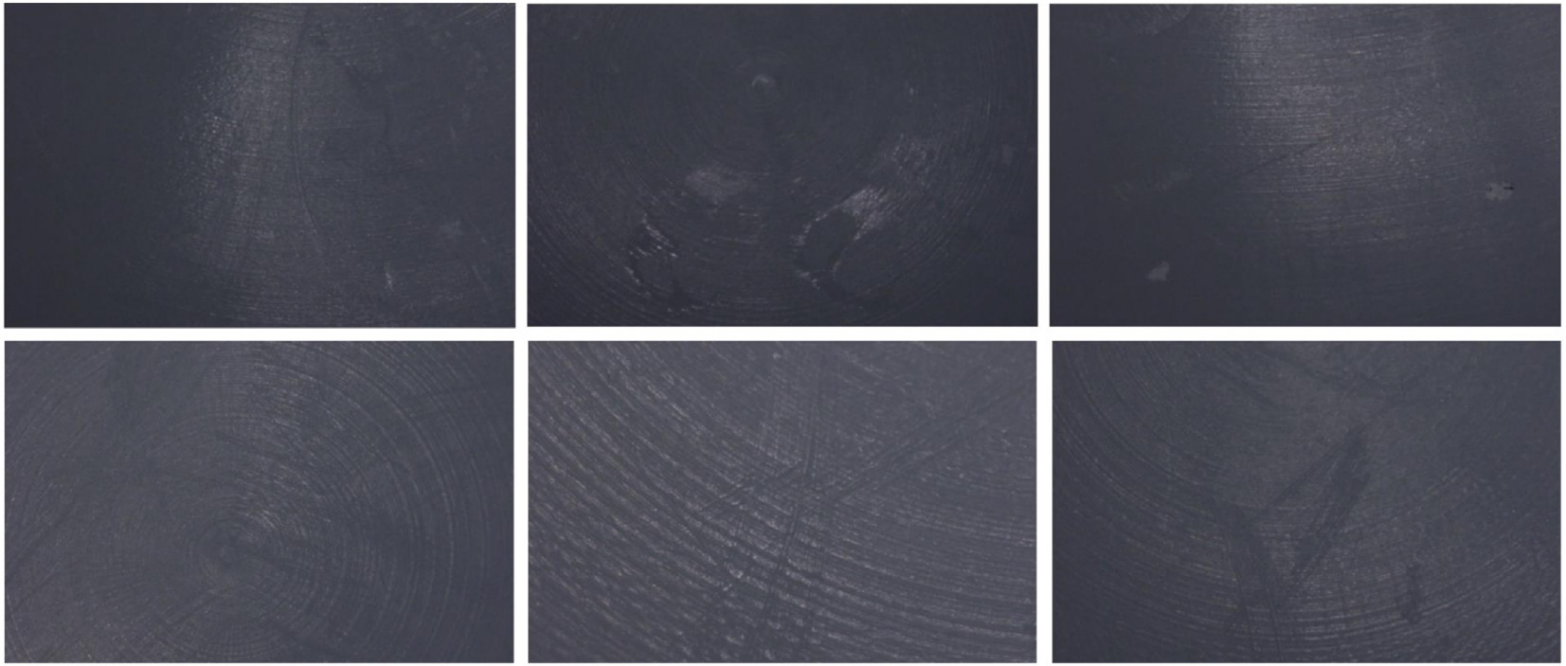
Examples of slight surface scratches identified on light microscopy (top 3 frames are liner 1, and bottom 3 frames are liner 4).

On light microscopy, subtle surface scratches were identified on all liners; representative examples are shown in Figure 2 below.

## Discussion

This study examined the impact of PMMA on polyethylene liners used in THA uniquely in a way that mimicked how the 2 materials could come into contact intraoperatively. The results of this study show that following exposure to PMMA during polymerization, isolated surface deposits on polyethylene liners can be seen in a dose-dependent manner. This suggests that protecting the liner’s articulating surface during cementing is necessary to prevent changes to the surface characteristics of the polyethylene.

When analyzing the liners, the control liner did not show significant evidence of surface deposits. In contrast, the liners that had 2 cc of cement applied all showed surface deposits. Additionally, only one of the liners that had just 1 cc of cement applied in absence of a femoral head had major surface changes. Furthermore, the 2 liners that had 2 cc of cement added had larger maximal surface deviations. Therefore, a threshold volume of cement appears to correlate with increased size of surface deposits. The liners with 1 cc of cement placed in the center of the cup with no external pressure and the liner that had 1 cc of cement with a head compressing the cement had no major differences in either maximal deviation or volumetric change. Thus, suggesting that increasing the surface area exposed to the same volume of cement does not influence the chance of creating surface deposits on the liner.

Overall, the results of this study show that the interaction between PMMA and polyethylene in liners used in orthopaedic surgery produces minimal gross deformation. However, the surface deposits on the polyethylene have the potential to act as third bodies to increase wear on the acetabular component in the artificial joint. Third-body wear is a process by which extraneous particles in the joint space accelerate deterioration of an artificial joint, and this has previously been associated with as many as 40% of cases of increased joint wear [12]. Factors that increase third-body wear include type of material, debris size, embedded location, and bearing surface with large metallic debris in the superolateral position in contact with a metal acetabular cup showing the highest rates of wear [[12], [13], [14]]. Previous literature has shown that bone cement particles that were 30 μm in size created minimal third body wear on XLPE liners [13] and that those containing zirconium increased surface roughening, which could lead to increased wear [15]. Only liners exposed to 2 cc of cement had on average deposits greater than 30 μm when compared to the control condition and thus may have had an increased chance of third body wear postoperatively. In this case, the surface deposits on the polyethylene liners have the potential to act as third bodies and, as they are present at time of surgery, could accelerate the process of third body wear when compared to joints in which wear is generated either due to (1) normal joint use over time or (2) third bodies that are created due to progressive joint degeneration [16].

The gravimetric analysis of the liners revealed an increase in volume postexposure for all liners except for liner 2. This increase could potentially be a result of cement debris remaining on the liner following cement removal, as the gravimetric approach approximates volume through mass. However, because the control also showed an increase in volume, this may not be entirely accurate. Damage during the removal of cement could potentially account for the loss of volume on liner 2.

The evidence found on light microscopy is of limited relevance as the scratches found were very superficial. In contrast to the isolated surface deposits, these areas were not evident on CMM scanning and therefore are unlikely to contribute to any meaningful extra joint wear that could result in joint failure.

### Limitations of this study

In this study, there are potential limitations to the CMM-generated surface scans. There are known artifacts when scanning hemispherical objects that typically cause deviations just off the center of the liner and toward the edges. Evidence of this central deviation can be seen in the heat map of all liners in Figure 1. There were no larger surface deposits identified in these areas and so these highlighted changes are unlikely an artifact of the software visualization of the scan. However, in liners without any clear surface deposits, these artifacts could have contributed to the maximal surface deviation recorded. Furthermore, the analysis does not extend to determining this composition of the deposits; therefore, it is not possible to determine whether they are PMMA or polyethylene from CMM analysis alone. The limited number of liners included in this study also makes it challenging to draw conclusions about differences in experimental groups. Despite this, the lack of gross deformation on all 6 liners and the presence of surface deposits on all but one of the experimental liners, and in all distinct experimental conditions when compared to the control show an overall pattern that is consistent.

### Future directions

As a continuation of this study, there is the potential to conduct further in vitro studies in addition to incorporating clinical participants. Increasing sample size and adding further experimental groups in vitro using different volumes of cement could allow further analysis of the critical threshold for development of surface deposits and elucidate any patterns in surface deposit location on the liners. Clinically, this could include following patients prospectively after a known interaction between cement and polyethylene liner bearing surfaces intraoperatively. Applicable cases could then be followed to confirm if this interaction increased long-term revision rates or quicker progression to implant failure due to increased wear and aseptic loosening. These investigations could also be expanded to include total knee arthroplasty, where cementing is commonly used.

## Conclusions

XLPE liners show minimal gross deformation in addition to isolated surface changes in a dose-dependent manner after coming into contact with highly exothermic PMMA. There is the potential for these surface deposits to act as third bodies to increase wear between a THA femoral head and polyethylene liner. Thus, care should be taken to avoid contact between large volumes of PMMA and polyethylene intraoperatively to prevent increased third-body wear.

## Conflicts of interest

A. Atrey is a consultant and receives support from ZB, S&N, DePuy, Biocomposites, and Stryker. He has also received financial support for educational project from ZB; all other authors declare no potential conflicts of interest.

For full disclosure statements refer to https://doi.org/10.1016/j.artd.2023.101184.

## Appendix A Supplementary data

Conflict of Interest Statement for Atrey

Conflict of Interest Statement for Hothi

Conflict of Interest Statement for Brillantes

Conflict of Interest Statement for Khoshbin

Conflict of Interest Statement for Thompson
