# Supplementary material for: A Geometric Analysis of Polyethylene Liners Exposed to Acrylic-based Bone Cement
Source: Arthroplast Today. 2023 Sep 19;23:101184. doi: 10.1016/j.artd.2023.101184 (PMC10517260; doi:10.1016/j.artd.2023.101184)
Supplement: Conflict of Interest Statement for Atrey [file mmc1.pdf]

INDIVIDUAL CONFLICT OF INTEREST STATEMENT

American Association of Hip and Knee Surgeons

(Adopted from the American Academy of Orthopaedic Surgeons disclosure statement)

The following form **must be filled out completely and submitted by each author (example, 6 authors, 6 forms). All items require a response. If there is no relevant disclosure for a given item, enter "*None*."**

Manuscript Title

1. Royalties from a company or supplier (The following conflicts were disclosed)
   NO

2. Speakers bureau/paid presentations for a company or supplier (The following conflicts were disclosed)
   NO

3A. Paid employee for a company or supplier (The following conflicts were disclosed)
   NO

3B. Paid consultant for a company or supplier (The following conflicts were disclosed)
   Consultant for ZB, S&N, DePuy, Biocomposites and Stryker

3C. Unpaid consultants for a company or supplier (The following conflicts were disclosed)
   NO

4. Stock or stock options in a company or supplier (The following conflicts were disclosed)
   NO

5. Research support from a company or supplier as a Principal Investigator (The following conflicts were disclosed)
   Support from ZB, S&N, DePuy, Biocomposites and Stryker

6. Other financial or material support from a company or supplier (The following conflicts were disclosed)
   Financial support for educational project from ZB

7. Royalties, financial or material support from publishers (The following conflicts were disclosed)
   NO

8. Medical/Orthopaedic publications editorial/governing board (The following conflicts were disclosed)
   NO

9. Board member/committee appointments for a society (The following conflicts were disclosed)
   NO

Each author must sign AND print or type his/her name, date and submit a separate form

In addition, one BLINDED Conflict of Interest form (no author names used) should be submitted per manuscript with all author disclosures.

Amit Atrey	[signature]	2nd of May 2022
Author Name (Print or Type)	Author Signature	Date
